# Síndrome de Landau-Kleffner: Actualidades en la Etiopatogenia y su Manejo

**DOI:** 10.31083/RN42643

**Published:** 2025-05-27

**Authors:** Alma D. Méndez-Álvarez, Karla P. Meneses-León, Dira B. Monsalvo-Soler, Alondra M. Morales-Segundo, Laura Gómez-Virgilio, Gustavo López-Toledo

**Affiliations:** ^1^Facultad de Medicina, Universidad Nacional Autónoma de México (UNAM), 04510 Ciudad de México, México; ^2^Coordinación de Investigación, Hospital Ángeles Lindavista, 07760 Ciudad de México, México; ^3^Departamento de Fisiología, Facultad de Medicina, Universidad Nacional Autónoma de México (UNAM), 04510 Ciudad de México, México

**Keywords:** afasia, diagnóstico molecular, electroencefalograma, epilepsia adquirida, proteína GRIN2A, terapia molecular dirigida, aphasia, molecular diagnosis, electroencephalogram, acquired epilepsy, GRIN2A protein, molecularly targeted therapy

## Abstract

El síndrome de Landau-Kleffner es una encefalopatía epiléptica y del desarrollo manifestado en la mayoría de los casos en pacientes pediátricos, caracterizado por la existencia de agnosia auditiva verbal, así como por la presencia de actividad epiléptica focal, bilateral y tanto focales como difusas, visualizada a través de la obtención de registros electroencefalográficos realizados durante el sueño. Es un síndrome poco común, de presentación variable, multifactorial y con etiología desconocida, aunque con un componente genético en algunos de los casos, mayoritariamente asociado a variantes del gen subunidad 2A del receptor ionotrópico de glutamato tipo N-metil-D-aspartato (NMDA), por sus siglas en inglés (*GRIN2A*), el cual codifica para la proteína del mismo nombre, perteneciente a una de las subunidades del receptor NMDA, y a su vez involucrada en diferentes procesos neurofisiológicos y cuyas modificaciones podrían asociarse a las manifestaciones clínicas observadas en los pacientes. Bajo este enfoque, el objetivo de la presente revisión es proponer un mecanismo fisiopatológico relacionado con una de las presentaciones clínicas de esta enfermedad, a través de la información publicada en los últimos años, contribuyendo al entendimiento de la patología, así como a su abordaje y manejo efectivos, dado que, al ser una enfermedad poco frecuente y compleja, tanto su diagnóstico como tratamiento implican un reto, lo que limita al paciente en el número de opciones terapéuticas y compromete su calidad de vida.

## Abstract (en)

Landau-Kleffner syndrome is a developmental epileptic encephalopathy that manifests mainly in pediatric patients, characterized by verbal auditory agnosia and focal, bilateral, and focal and diffuse epileptic activity, visualized through electroencephalographic recordings performed during sleep. It is a rare syndrome with a variable, multifactorial presentation and unknown etiology, although it has a genetic component in some cases. It is often associated with variants of the glutamate ionotropic receptor N-methyl-D-aspartate (NMDA) type subunit 2A (*GRIN2A*) gene, which encodes an NMDA receptor subunit of the same name that is involved in various neurophysiological processes. Modifications to this receptor could be associated with the clinical manifestations observed in patients. This review proposes a pathophysiological mechanism related to one of the clinical presentations of this disease, using information published in recent years, and contributes to the understanding of its pathology and the improvement of its management. This syndrome is a rare and complex disease; both its diagnosis and treatment are challenging, limiting patients’ therapeutic options and compromising their quality of life.

## 1. Introducción

El síndrome de Landau-Kleffner es una rara condición en la que los niños pierden la habilidad para hablar y entender el lenguaje, caracterizado por una actividad anormal en el cerebro durante el sueño, acompañado en algunos casos de epilepsia, ocasionando problemas en el comportamiento, así como afecciones graves en la interacción e integración con la sociedad. Hasta el momento no se cuenta con suficiente evidencia acerca de las causas y el desarrollo de esta enfermedad y, aunque se ha reportado que tiene un componente genético, son pocos los casos asociados a la variación de uno o varios genes en particular, sin embargo, el comprender los procesos fisiopatológicos asociados a estas variaciones en el genoma, podría conducir al desarrollo de terapéuticas efectivas que permitan una mejor calidad de vida al paciente. El objetivo del trabajo es exponer el panorama actual de la enfermedad desde su definición hasta la propuesta de un posible mecanismo molecular–celular–funcional que explique una de las presentaciones clínicas relacionadas a este síndrome.

## 2. Desarrollo

### 2.1 Síndrome de Landau-Kleffner: Definiciones y Etiología

La encefalopatía epiléptica (EE) es un concepto que describe aquellas afecciones, ya sean graves deterioros cognitivos y conductuales, en los que la actividad epiléptica en sí misma contribuye más allá de lo que se esperaría de alguna otra patología subyacente [1]. Con una etiología variada, la cual puede ser de tipo genética, adquirida o ambas, y aplicable a todas las edades [2, 3].

En la EE, la abundante actividad epileptiforme interfiere con el desarrollo, ya sea superpuesta al desarrollo normal o a un retraso en el desarrollo preexistente, sin embargo, en algunas condiciones, el impacto en el desarrollo parece ser independiente de la EE, razón por la cual, la clasificación de las epilepsias de la Liga Internacional Contra la Epilepsia (ILAE, por sus siglas en inglés), en el 2017, introdujo el término encefalopatía epiléptica y del desarrollo (DEE, por sus siglas en inglés) para reconocer que ambos aspectos pueden estar desempeñando un papel en la presentación clínica de la encefalopatía. La ILAE sugiere que dicho término sea utilizado cuando sea apropiado, es decir, encefalopatía del desarrollo (DE, por sus siglas en inglés) cuando se presente deterioro del desarrollo sin actividad epiléptica asociada; encefalopatía epiléptica donde no exista retraso en el desarrollo y encefalopatía epiléptica y del desarrollo donde ambos factores juegan un papel en la enfermedad [4].

Las DEEs son un grupo de trastornos epilépticos raros que ocurren en edad pediátrica y cuya etiología puede deberse a causas genéticas como no genéticas. En el caso de las causas genéticas, éstas se han asociado a variantes de genes que codifican proteínas sinápticas y canales iónicos. Siendo en la mayoría de los casos altamente resistentes a fármacos, muestran regresión del desarrollo y presentan anomalías graves en el electroencefalograma (EEG) [5]. Una de las anomalías presentadas en el EEG es el patrón de activación marcada de picos y ondas durante el sueño, asociado a una regresión en varios dominios incluidos el comportamiento, el lenguaje, la cognición, la atención, la interacción social y las habilidades motoras. El síndrome de Landau-Kleffner es una DEE que presenta esta anomalía en el EEG y cuyos síntomas principales son la afasia aguda o subaguda con incapacidad para reconocer, procesar o interpretar sonidos verbales o no verbales, o ambos [3]. William Landau y Frank Kleffner caracterizaron este síndrome en 1957, cuya peculiaridad comienza con la agnosia auditiva, además de la aparición en la dificultad del habla en niños previamente sanos, así como la probabilidad de un 70% de desarrollar convulsiones [6, 7].

La etiología del síndrome de Landau-Kleffner (LKS) sigue siendo desconocida, aunque no existe una base genética clara, alrededor del 20% de las personas con este síndrome presentan variantes en el gen subunidad 2A del receptor ionotrópico de glutamato tipo N-methyl-D-aspartate (NMDA), por sus siglas en inglés (*GRIN2A*) [8]. También se señala una posible implicación inmunológica, ya que se ha demostrado la presencia de niveles elevados de autoanticuerpos dirigidos contra el factor neurotrófico derivado del cerebro (BDNF) en sujetos con el LKS en comparación con controles sanos [6]. Además, se ha sugerido que una sinaptogénesis anormal en regiones importantes para el procesamiento del lenguaje durante el desarrollo neuronal tiene como consecuencia alteraciones persistentes del lenguaje, situación característica en el LKS [9].

### 2.2 Epidemiología: Incidencia y Prevalencia

El LKS se describe como un síndrome con series de casos esporádicos o limitados. La prevalencia e incidencia son difíciles de valorar, sin embargo, una estimación epidemiológica realizada en Japón concluyó que la incidencia de niños con el LKS de entre 5 y 14 años fue de aproximadamente de 1 en un millón mientras que la prevalencia de pacientes entre 5 y 19 años fue de 1 entre aproximadamente 300.000 a 410.000 [10]. Por otro lado, los casos descritos en la literatura muestran que los hombres son más afectados que las mujeres con una relación 2:1 [6], mientras que la edad promedio fluctúa entre los 18 meses y 14 años, aunque presenta una mayor incidencia entre los 3 y 7 años [8].

### 2.3 Manifestaciones Clínicas y Diagnóstico

El LKS se describe como una entidad heterogénea, presentando una considerable variación en la edad de inicio, las características de la afasia, las anomalías asociadas al EEG, así como los trastornos cognitivos y conductuales [11]. Una de sus manifestaciones distintivas es la afasia, la cual está presente en todos los pacientes. En este sentido, el 50% de los casos debuta con afasia verbal auditiva, sin presencia de alteraciones auditivas, con progreso a una afasia expresiva y dificultad para el procesamiento o interpretación de los sonidos verbales y/o no verbales. En el caso de niños, la clínica puede ser expresada como balbuceo, neologismos, perseverancia verbal o mutismo, mientras que en pacientes jóvenes se manifiestan dificultades expresivas acompañadas de un lenguaje limitado y alterado [6, 12].

Respecto a las alteraciones en el comportamiento asociadas al LKS, los pacientes muestran irritabilidad, hiperactividad, impulsividad y dificultad para la interacción social. Además, puede acompañarse de déficit de atención, distracción, labilidad emocional, ansiedad, depresión, trastornos del sueño, alteración de la memoria de trabajo y en algunos casos presencia de hipersensibilidad a sonidos [12]. No todos los pacientes con el LKS presentan crisis convulsivas, y quienes cursan con ellas se manifiestan como crisis motoras parciales, siendo las más comunes presentándose entre el 75–83% de los casos [12]. También pueden cursar con crisis clónicas generalizadas y crisis de ausencia atípicas, que incluyen comportamientos como parpadeo repetitivo, gestos de masticación, chasquidos o movimientos bruscos de labios. Cabe señalar que los pacientes pueden presentar afasia grave y no asociar con crisis convulsivas [6].

En cuanto a la actividad eléctrica del EEG, ésta se encuentra generalizada e incrementada durante la fase del sueño en la que no se producen movimientos oculares rápidos o sueño no sueño de movimientos oculares rápidos (REM) (REM, por sus siglas en inglés) con un patrón de picos-ondas de forma casi continua y una actividad de fondo que suele ser normal. En relación con las anomalías asociadas al EEG, todos los pacientes presentan picos y ondas bilaterales en más del 85% del sueño no REM, mientras que, durante el sueño REM, esta actividad epiléptica se puede interrumpir, disminuir o remitir parcialmente [6, 12, 13].

Para poder diferenciar de otros trastornos del lenguaje, el LKS requiere de una evaluación integral basada en anamnesis, evaluaciones clínicas, estudios electroencefalográficos y de neuroimagen, así como de pruebas moleculares. Un resumen de lo reportado para este síndrome en cada evaluación o prueba se describe en la Tabla 1 (Ref. [6, 13, 14, 15]). En relación con las pruebas moleculares, el porcentaje de pacientes que tienen asociada una variante genética a dicha patología no es muy grande, pero la determinación de modificaciones en el genoma permite otorgarles un diagnóstico preciso, por lo que la identificación de variantes génicas podría explicar el fenotipo de enfermedad, al menos en esos casos.

**Tabla 1.** **Pruebas o evaluaciones reportadas para el diagnóstico del LKS**.

Prueba diagnóstica	Técnica	Descripción	Limitaciones	Refs.
Evaluación clínica		∙ Alteraciones agudas o graduales del lenguaje, trastornos del comportamiento como hiperexcitabilidad e hiperactividad en niños con un desarrollo previamente normal.	∙ Solamente un 15% de los pacientes presenta un retraso en el habla antes de la regresión.	[6, 13, 14]
		∙ Puede asociarse o no a convulsiones.	∙ No todos los niños presentan convulsiones clínicamente manifiestas.	
Neuroimagen	Magnetoencefalografía (MEG)	∙ Registros de picos originados alrededor de la corteza auditiva izquierda, picos secundarios originados en las áreas perisilvianas, temporooccipital y parietooccipital ipsilaterales. La técnica es de utilidad para la localización de focos epilépticos en el mapeo de funciones corticales, así como la evaluación de la actividad epiléptica intersticial.	∙ La MEG es altamente sensible al movimiento, por lo que implica un desafío ya que si el paciente se encuentra en movimiento puede ocasionar una deficiente calidad en la obtención de los datos dificultando su interpretación.	[15]
			∙ Debe tener una interpretación conjunta con otros estudios.	
	Tomografía computarizada por emisión de fotón único (SPECT, por sus siglas en inglés) y Tomografía de emisión de positrones (PET, por sus siglas en inglés)	∙ La evaluación con PET es utilizada principalmente para poder visualizar áreas con actividad epileptogénica en regiones corticales. En el caso de pacientes con el LKS se ha observado una actividad reducida en regiones corticales. Por otro lado, la evaluación con SPECT muestran hipoperfusión dentro y sobre el lóbulo frontal izquierdo principalmente.	∙ Los hallazgos que puedan presentarse no explican la causa ni la presentación clínica del LKS, por lo tanto, se requiere interpretación conjunta con otras pruebas.	[15]
	Resonancia magnética	∙ Se ha observado reducción en el volumen de áreas del cerebro implicadas en el desarrollo del lenguaje.	∙ Solamente permite excluir otros diagnósticos como tumores u otras lesiones estructurales.	[6]
Electroencefalograma (EEG)		∙ Los picos, las espigas y las ondas, tanto agudas como lentas, son manifestaciones frecuentes en los hallazgos del EEG en pacientes con el LKS y su aparición es en función de la etapa de la enfermedad de cada paciente. Los trazados del EEG focales anormales se pueden detectar en cualquier área cortical y pueden o no ser simétricos. Aunque se ha postulado que hay un predominio temporal izquierdo de la actividad cortical.	∙ Las anomalías que frecuentemente se pueden encontrar no se detectan de primera instancia, se observan hasta meses después de la presentación inicial en algunos casos.	[14, 15]
			∙ Las anomalías presentes en el EEG no siempre son un reflejo de sus síntomas verbales auditivos.	
Audiometría		∙ En etapas agudas a subagudas de la enfermedad, los hallazgos audiométricos de tono puro en ocasiones muestran elevaciones leves en los umbrales auditivos.	∙ Con la prueba, algunos pacientes con el LKS no pueden localizar auditivamente las fuentes de sonido.	[15]
		∙ La secuela a largo plazo parece deberse a la discriminación anormal de palabras. Cuando los pacientes con el LKS tienen agnosia auditiva no verbal como complicación, a menudo se observan anomalías en las puntuaciones de las pruebas de discriminación de sonidos ambientales. La prueba Token suele ser útil para discernir la capacidad de un paciente para comprender palabras, frases y oraciones.	∙ Si los pacientes pueden discriminar diferencias en la intensidad del sonido, generalmente no pueden discriminar diferencias de intervalos de tiempo.	
Pruebas genéticas	Secuenciación o Microarreglos	∙ En pacientes con antecedentes de historia familiar de epilepsia o de dificultades en el lenguaje se puede realizar secuenciación genética para determinar variantes en el gen *GRIN2A*, y otros genes asociados.	∙ No forma parte de los métodos diagnósticos habituales	[6, 14]
			∙ Las técnicas solo se encuentran disponibles en centros especializados.	

*GRIN2A*, subunidad 2A del receptor ionotrópico de glutamato tipo N-methyl-D-aspartate (NMDA), por sus siglas en inglés; LKS, síndrome de Landau-Kleffner; Refs, Referencias.

### 2.4 Fisiopatología

Dada la heterogeneidad del LKS, además de su baja frecuencia, resulta complicado establecer una fisiopatología, sin embargo, abordándola desde la perspectiva de trastornos similares y realizando una aproximación mediante enfoque genético podría dilucidarse algún mecanismo aplicable a dicho síndrome. El siguiente apartado pretende contextualizar al LKS desde el espectro de epilepsia–afasia al que pertenece, hasta llegar a las variantes de un gen fuertemente asociado a este síndrome y su afectación a nivel clínico–funcional.

Se definen a las epilepsias focales, inicialmente denominadas idiopáticas (IFE, por sus siglas en inglés), como enfermedades que ocurren durante períodos críticos del desarrollo, generalmente durante la infancia y cuya forma más frecuente es la epilepsia rolándica (RE, por sus siglas en inglés) que, junto al síndrome de Landau-Kleffner y la encefalopatía epiléptica relacionada con el estado epiléptico durante el síndrome del sueño lento (ESES, por sus siglas en inglés), forman parte de un espectro de epilepsias y encefalopatías epilépticas infantiles con deterioro cognitivo, conductual y del habla designado como espectro de epilepsia–afasia (EAS, por sus siglas en inglés). Dichas patologías comparten la asociación de convulsiones con descargas paroxísticas del EEG activadas durante el sueño con déficits neuropsicológicos y conductuales adquiridos [16]. Respecto a las causas de estas patologías, se ha manejado el supuesto de que, a diferencia de las epilepsias generalizadas, la mayoría de las epilepsias focales son causadas por agentes ambientales más que por genéticos [17, 18, 19]. Sin embargo, los familiares de pacientes con RE, LKS o ESES muestran un mayor riesgo de epilepsia que el resto de la población [20, 21, 22].

Dados los avances tanto en citogenética molecular como en la secuenciación de ácidos nucleicos de próxima generación, se han podido determinar alteraciones correspondientes a genes asociados con el espectro del trastorno autista o implicados en alteraciones del habla, siendo notable dada la asociación del LKS y ESES con manifestaciones similares al autismo y trastornos del lenguaje [20]. En este sentido, se han reportado alteraciones del gen *GRIN2A*, el cual codifica para una subunidad del receptor de glutamato NMDA (NMDAR), en sujetos con trastornos graves del desarrollo neurológico y se ha indicado que los pacientes con variantes en dicho gen muestran disartria, dispraxia o disfasia, hipernasalidad, prosodia anormal, además de manifestar regresión del lenguaje y daño cognitivo [23, 24]. Lo anterior muestra la importancia de este gen en el procesamiento del lenguaje a nivel clínico, pudiéndose explicar desde una perspectiva anatómica, ya que áreas como la corteza prefrontal, el estriado, el hipocampo y el tálamo expresan la proteína GluN2A o GRIN2A (anteriormente conocida como NR2A), codificada por el gen *GRIN2A* [25, 26, 27, 28]. Por otro lado, las consecuencias neurofisiológicas de las alteraciones a este gen se pueden evaluar a través del bloqueo de los receptores que contienen GRIN2A, los resultados de dicho bloqueo muestran un aumento de la potencia gamma y reducción de su modulación por las bajas frecuencias entre las que se implican a las categorías lingüísticas [29, 30]. La modulación de alta frecuencia por ritmos más bajos al parecer son la base de una comunicación interregional en donde se busca establecer aquellas dinámicas espaciotemporales asociadas con las deficiencias lingüísticas reportadas en pacientes con el LKS [8]. Respecto a los anomalías relevantes del EEG en el LKS, existen efectos de las descargas epileptiformes interictales (IED, por sus siglas en inglés) durante el sueño sobre el lenguaje, lo anterior se sabe ya que se ha observado que las habilidades lingüísticas de los pacientes con el LKS varían en proporción a la gravedad de los IED nocturnos, pudiéndose ligar a las variantes en el gen *GRIN2A* [31, 32], quien ha destacado como un gen candidato para el patrón del EEG en ESES [21], y se ha señalado que en el LKS la actividad epiléptica del EEG altera la arquitectura del sueño, interfiriendo con la consolidación de la memoria [33], coincidiendo con la hipótesis de la homeostasis sináptica, en la que se establece que los cambios plásticos culminan en un incremento de la fuerza sináptica en varios circuitos, lo que resulta poco sostenible debido al alto consumo de energía de sinapsis más fuertes, y es aquí en el sueño en donde se busca reducir la fuerza sináptica acumulada en la vigilia [34], además de que en el EEG la actividad de onda lenta es un marcador de normalización sináptica presentada en el sueño [33], por lo que la actividad epiléptica focal prolongada durante el sueño interfiere con los mecanismos para generar actividad de onda lenta [35], perjudicando cambios plásticos asociados con la cognición y el aprendizaje, en el que la gravedad es proporcional al grado de descargas epileptiformes, con interferencia negativa en las funciones plásticas del sueño y la renovación sináptica [36, 37, 38].

Dada la importancia de la subunidad GluN2A del receptor NMDA, resulta necesario contextualizar a GRIN2A desde su estructura y posteriormente desde función, para después relacionarla con las variantes reportadas en los pacientes con el LKS y cómo influyen en la fisiopatología y fenotipo de la enfermedad.

Los receptores NMDA son canales iónicos activados por ligando, permeables a cationes como Na^+^, K^+^ y Ca^2+^, cuya estructura molecular se encuentra constituida por dos subunidades GluN1, las cuales se unen a glicina, y dos subunidades GluN2/3, que se unen a glutamato (GluN2A, GluN2B, GluN2C, GluN2D, GluN3A y GluN3B) [39]. Todas las subunidades comparten una estructura similar:

∙ Contienen un dominio amino terminal extracelular (NTD, por sus siglas en inglés), con sitios de unión para moduladores alostéricos.

∙ Un dominio de unión a ligando extracelular bilobulado (LBD, por sus siglas en inglés).

∙ Un dominio transmembrana formador de poros (TMD, por sus siglas en inglés) del canal que comprende cuatro segmentos hidrófobos (M1–4).

∙ Un dominio carboxilo terminal intracelular (CTD, por sus siglas en inglés), que se asocia con proteínas postsinápticas que median señalización intracelular.

El NMDAR se caracteriza por una cinética de corriente relativamente lenta, un bloqueo dependiente de voltaje por parte del Mg^2+^ extracelular y una alta permeabilidad al Ca^2+^. Debido a su patrón de expresión, se encuentra implicado en el neurodesarrollo, la plasticidad, así como en el aprendizaje y funciones cognitivas superiores [40].

Respecto al espectro de fenotipos asociados a modificaciones en el gen *GRIN2A*, en el 2012 Lesca, identificó un paciente con el LKS portador de una microdeleción que altera al gen *GRIN2A* [20]. Un año después, el mismo grupo identificó variantes en el gen *GRIN2A* en alrededor del 20% de individuos que presentaron un fenotipo clínico de LKS [41]. Otro estudio del mismo año describió en cuatro familias la segregación autosómica dominante de la microdeleción de *GRIN2A* en EAS en las que se incluye al LKS [42], y se demostró que casi el 8% de todos los pacientes con epilepsia focal idiopática tenían variantes en el gen *GRIN2A*, de entre los cuales el 13% correspondían al LKS [43]. En cuanto al tipo de variantes, se evidenció que en el caso del LKS, había una menor proporción de variantes sin sentido, asociadas a fenotipos benignos en otros tipos de EAS, mientras que la mayor proporción correspondían a truncaciones y se relacionaron con fenotipos más severos en el LKS [43]. Sin embargo, si se analizan todos los casos publicados en 2013, la proporción cambia respecto al LKS, ya que la proporción es de aproximadamente 3:1 siendo las variantes sin sentido las de mayor proporción respecto a las truncaciones, independientemente de la gravedad clínica [41, 42, 43]. Un estudio reciente reportó una variante de novo patogénica del gen *GRIN2A*, la cual no había sido reportada previamente (c.2094T>G (p.Y698*)), concluyendo que dicha variante comprometía el dominio de unión a ligando, además de provocar una pérdida de la función en el NMDAR [44]. Asimismo, se identificó en pacientes con el LKS una variante no reportada en el gen *GRIN2A*, mediante la técnica de secuenciación de exoma, reportando variantes de genes candidatos que podrían estar involucrados en el desarrollo de esta patología como proteoglicano 2 del heparán sulfato (por sus siglas en inglés) (*HSPG2*), nidogen-2 (*NID2*), subunidad alfa 5 de la laminina (por sus siglas en inglés) (*LAMA5*), subunidad alfa 1 de la laminina (por sus siglas en inglés) (*LAMA1*), neurocan (*NCAN*), reelina (*RELN*), subunidad beta 5 de la integrina (por sus siglas en inglés) (*ITGB5*), subunidad alfa 9 del canal dependiente de voltaje de sodio (por sus siglas en inglés) (*SCN9A*), miembro 3 de la familia de transportadores de solutos 30 (por sus siglas en inglés) (*SLC30A3*), receptor 2 de efrina tipo B (por sus siglas en inglés) (*EPHB2*) y receptor 5 de efrina tipo A (por sus siglas en inglés) (*EPHA5*) [22].

A continuación, se describe el análisis funcional *in vitro*, reportado para 2 variantes sin sentido en el gen *GRIN2A* identificadas en niños con el LKS, el cual fue reportado en 2017 para éstas y otras 8 variantes presentes en otros trastornos pertenecientes al espectro de las EAS y cuyo criterio de selección se realizó de acuerdo a puntuaciones de Agotamiento Dependiente de Anotación Combinada (CADD, por sus siglas en inglés) asignadas a todas las variantes identificadas en las cohortes del 2013 [45]. Las puntuaciones CADD permiten crear predicciones de carácter nocivo basadas en métricas como la conservación y estructura de proteínas, cuyo valor mayor de 20 ubicaría a cada variante dentro del 1% de las variantes más nocivas [46], además de localizarse dentro de los dominios NTD, LBD o en el poro, sitios en donde existe el pronóstico de efectos funcionales más graves comparado con el dominio CTD. Las 10 variantes de este reporte permiten analizar variantes presentes en diferentes dominios funcionales, con diferentes patrones de herencia, diferentes valores CADD, así como resultados en la enfermedad, tal y como se aprecia para los casos del LKS representados en la Tabla 2 (Ref. [41, 43]).

**Tabla 2.** **Información clínica y genética de las variantes en el gen *GRIN2A* detectadas en niños con LKS**.

Variante sin sentido	Cambio aminoacídico	Dominio afectado	Cambio estructural	Patrón de herencia/Segregación	Puntos CADD	Resultado en fenotipo	Ref.
c.692G >A	C231Y	NTD	Si, hipótesis	Materna/Si	26,7	Severo: epilepsia, retraso en habla, motor y cognitivo	[43]
c.2797G >A	D933N	CTD	Desconocido	Paterna/No	28	Regresión de lenguaje, déficit de atención	[41]

NTD, dominio amino terminal extracelular (por sus siglas en inglés); CTD, dominio carboxilo terminal intracelular (por sus siglas en inglés); CADD, agotamiento dependiente de anotación combinada (por sus siglas en inglés).

Los resultados de los análisis *in vitro* realizados para estas variantes mostraron que la variante C231Y genera un fenotipo de potencia agonista y de expresión de la subunidad GluN2A del NMDAR en la superficie celular significativamente reducidos, lo que coincide a nivel clínico con un LKS más severo [45]. La reducción en la potencia del glutamato y la glicina se debe al cambio de cisteína a tirosina en una cavidad hidrofóbica del dominio NTD, provocando un plegamiento incorrecto de las proteínas, con una consecuente alteración en la unión de los agonistas, además de la degradación de las proteínas observado en una menor expresión de la subunidad del receptor [45]. Tanto la disminución en la expresión como la falta de respuesta a los agonistas puede causar redes cerebrales hiperexcitables mediante varios mecanismos, por ejemplo, la actividad del NMDAR que contiene Glu2NA es fundamental en el desarrollo y mantenimiento de la función gabaérgica de las interneuronas parvalbúmina positivas, por lo que una reducción de la función de Glu2NA en estas interneuronas podría provocar un funcionamiento deficiente de la red inhibidora [47]. Por otro lado, durante la etapa postnatal, existe un incremento en la expresión de las subunidades GluN2A, sustituyendo a las GluN2B en la sinapsis, lo que resulta en un equilibrio entre plasticidad y estabilidad sináptica, necesarias para la maduración del aprendizaje asociativo, amortiguando la hiperexcitación debida a la presencia de una mayor información sensorial [48]. Por lo tanto, una menor expresión de Glu2NA en esta etapa crearía una red inhibidora subdesarrollada, con potencial de desarrollar actividad epiléptica. Finalmente, respecto a la variante D933N, no se observó efecto alguno en la función del NMDAR [45], pero se sabe que el dominio CTD de Glu2NA se acopla a cascadas de señalización postsináptica o al andamiaje, a través de modificaciones postraduccionales [49].

**Fig. 1.**
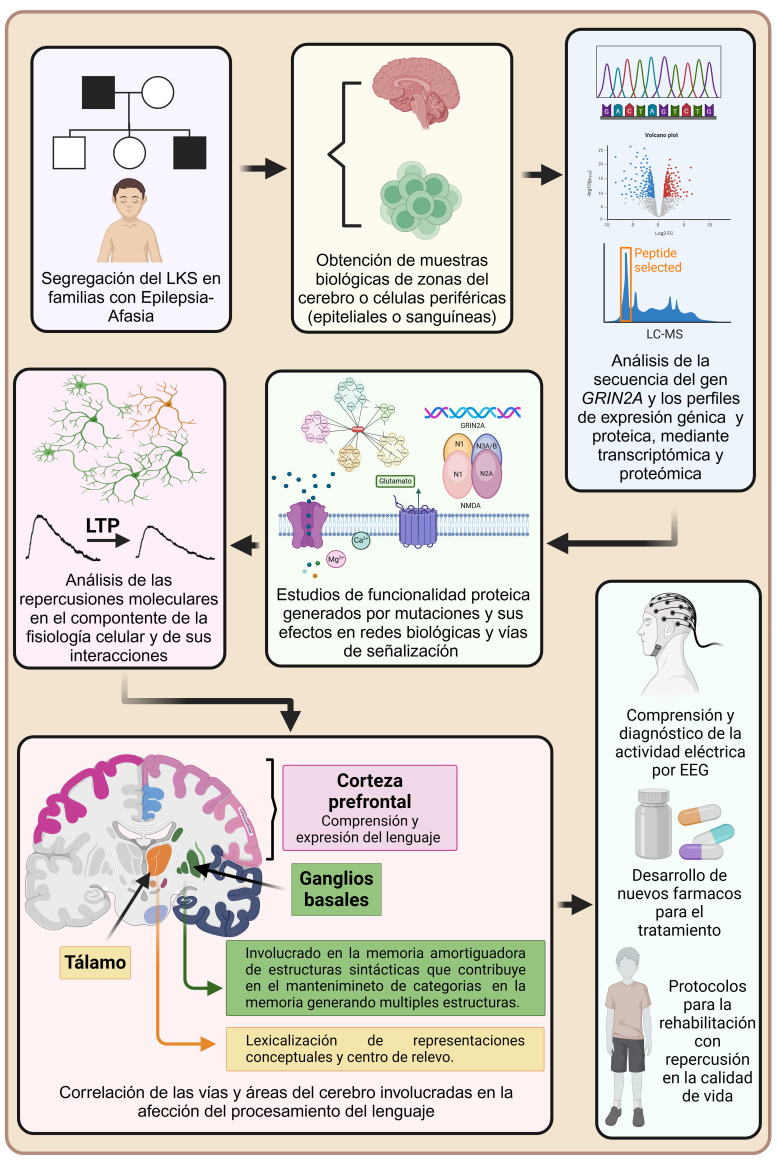
**Relación entre las causas genéticas del LKS y los posibles mecanismos fisiopatológicos de la enfermedad representados desde el abordaje experimental**. El seguimiento de familias candidato por situaciones de epilepsia-afasia entre las que se encuentra el LKS y su asociación a variantes en el gen *GRIN2A* se pueden segregar a lo largo de generaciones. La identificación de variantes en este gen requiere de la obtención de muestras cerebrales o periféricas ya sean en estado embrionario o adulto para la secuenciación del propio gen o de genes relacionados al equilibrio de la plasticidad y estabilidad sináptica, así como para el estudio de perfiles de expresión génica y proteica que permitan vislumbrar la interacción entre moléculas y vías de señalización importantes para la memoria y el aprendizaje, tales como las relacionadas al control de la excitabilidad neuronal y la potenciación a largo plazo. Además, el estudio de GRIN2A como elemento estructural del NMDAR y a su vez este último posicionado en poblaciones específicas de interneuronas encargadas del control inhibitorio de regiones cerebrales a través de diversas vías que controlan la actividad eléctrica cortical, son cruciales para el mantenimiento de información fonémica y silábica relacionada al procesamiento del lenguaje, permitiendo diagnósticos más certeros a través de la actividad eléctrica cortical analizada por EEG, el desarrollo de terapéuticas farmacológicas dirigidas a los pacientes, así como de estrategias de rehabilitación eficientes que repercutan en la calidad de vida del paciente. NMDAR, receptor de N-metil-D-aspartato; LTP, potenciación a largo plazo (por sus siglas en inglés). Creada en BioRender. López-Toledo, G. (2025) https://BioRender.com/gg1pmx2.

De acuerdo con lo descrito previamente, las variantes del gen *GRIN2A* pueden provocar ganancia, pérdida o ningún cambio en la función electrofisiológica del NMDAR dependiendo del tipo de variante como de su ubicación [23]. Por ejemplo, las variantes en el dominio CTD pueden no causar alteraciones electrofisiológicas o manifestar fenotipos leves, sin embargo, éstos últimos se presentan si se asocian con una disminución de la corriente sináptica excitadora espontánea, interacciones del NMDAR interrumpidos y en general una función sináptica reducida [50]. Sin embargo, no todos los cambios funcionales afectan de la misma manera la actividad del NMDAR, por lo que se desconoce el impacto funcional de muchas variantes del gen *GRIN2A*, motivo por el que, sugerimos un posible mecanismo fisiopatológico asociado al LKS desde la perspectiva genética, el cual se representa en la Fig. 1.

### 2.5 Tratamiento

El manejo del LKS abarca distintas intervenciones que incluyen medidas farmacológicas, modificaciones a la dieta, intervenciones quirúrgicas y técnicas de rehabilitación con tratamiento lingüístico cognitivo y terapia conductual (ver Tabla 3, Ref. [12, 13, 14, 51, 52, 53, 54, 55, 56]), sin embargo, hasta el momento, no existe un protocolo de intervención que incluya una muestra significativa de pacientes con este síndrome debido a la rareza y variedad en la presentación de la enfermedad [57]. A pesar de ello, el enfoque principal del tratamiento se centra en buscar la regularización de los signos y síntomas, siendo el habla y el comportamiento los aspectos desafiantes a tratar. A la fecha, las intervenciones no logran mejorar esos aspectos de la patología, en el que se busca conseguir que el paciente pueda llevar a cabo su vida con normalidad [58].

**Tabla 3.** **Tratamientos reportados para el manejo del LKS**.

Tipo de tratamiento	Tratamiento	Población objetivo	Mecanismo de acción	Efectos adversos (EA)	Resultados	Ref.
Farmacológico	Esteroides	Casos severos de LKS	∙ Su uso se basa en la evidencia de la asociación de las convulsiones con inflamación y viceversa. Además de la posible interacción de los esteroides con el receptor de GABA, suprimiendo una posible hiperexcitabilidad.	∙ Suelen ser bien tolerados. Los EA asociados a tratamiento de corto plazo incluyen problemas metabólicos, infecciones y efectos psicológicos. Los EA asociados a tratamiento de largo plazo incluyen retraso del crecimiento, osteoporosis, psicosis y atrofia cerebral.	∙ Parcialmente efectivos en alrededor del 50 al 70% de los casos, en dosis altas, régimen diario durante 4 a 6 semanas, continuando de forma intermitente hasta mantener recuperación.	[14, 51]
	Anticonvulsivos	Casos típicos de LKS	∙ Bloqueo de canales de sodio dependientes de voltaje o disminución de aminoácidos como el aspartato.	∙ Sedación significativa, coordinación reducida y problemas de comportamiento.	∙ Eficaces para tratar convulsiones, pero no para recuperar el lenguaje y la función cognitiva. Efectivos en un 30–50%.	[14, 52, 53]
Dieta cetogénica	Dieta cetogénica	Casos de LKS que no responden a tratamiento, terapia alternativa a estado epiléptico refractario	∙ Cetosis estimula conversión de glutamato a glutamina, así como la conversión de glutamina a GABA, disminuyendo susceptibilidad a epilepsia.	∙ 10% de los pacientes pueden tener efectos gastrointestinales. A largo plazo se ha reportado deshidratación, formación de cálculos renales y daño hepático en algunos pacientes y miocardiopatía en casos más graves. La mayoría de estos EA son transitorios.	∙ De acuerdo con algunos reportes, los pacientes muestran entre un 40 y 50% de posibilidades de reducir en al menos un 50% las convulsiones.	[52, 54]
Quirúrgico	Transecciones subpiales múltiples (MST, por sus siglas en inglés)	Casos de LKS refractarios al tratamiento farmacológico. Útil para pacientes con retraso en desarrollo neurológico, focos epileptógenos focales y EEG con un patrón hemi-ESES	∙ La técnica quirúrgica interrumpe selectivamente las fibras intracorticales horizontales asociadas a las descargas epileptiformes en las circunvoluciones temporales superior y media y en el área suprasilviana sustancial.	∙ Hay una mayor probabilidad de tener una regresión total en comparación con otros tratamientos.	∙ Mejora en la actividad y el comportamiento de las convulsiones, pero la recuperación de la función del lenguaje es variable.	[12, 55]
Terapia de rehabilitación	Logopedia	Casos de LKS aun teniendo otro tratamiento, ya que pacientes muestran déficits en procesamiento del lenguaje.	∙ Las intervenciones incluyen lenguaje de señas, diario de imágenes con secuencia dentro del aula y entrenamiento auditivo. El entrenamiento del habla y lenguaje enfocado en mejorar afecciones verbales auditivas que provocan dificultades del habla.	∙ Más que EA, evidencia sugiere que este tipo de terapia no mejora la agnosia verbal auditiva.	∙ Los trabajos existentes muestran mejoría en la comunicación empleada para la vida diaria de los pacientes.	[13, 56]

GABA, ácido gamma-aminobutírico (por sus siglas en inglés); ESES, estado epiléptico durante el síndrome del sueño lento (por sus siglas en inglés).

## 3. Conclusiones

El acceso a nuevas tecnologías, como la secuenciación, han permitido la identificación de variantes en el gen *GRIN2A* clínicamente relevantes en pacientes con el LKS. Diversos reportes de variantes de este gen se han asociado a otras enfermedades como la encefalopatía epiléptica con activación pico-onda durante el sueño (EE-SWAS) y encefalopatía epiléptica del desarrollo con activación de punta-onda durante el sueño (DEE-SWAS, por sus siglas en inglés) así como el síndrome de West. Tomando en cuenta la variabilidad individual en este gen, así como el medio ambiente y el estilo de vida de cada paciente se deben buscar mejores manejos y opciones terapéuticas para cada trastorno. En este sentido, desde la perspectiva genética, el LKS es una patología cuyo enfoque terapéutico es susceptible a un manejo individualizado orientado a la causa molecular de la enfermedad, permitiendo mayor precisión, efectividad y seguridad, un reto que hasta el momento permanece vigente dada la variabilidad en su presentación y rareza en cuanto al número de casos reportados.

En resumen, se ha contrastado el panorama actual en el cual se encuentra el LKS, los avances respecto a su etiopatogenia, diagnóstico y tratamiento, considerando aquella información fundamental y necesaria para ofrecer un contenido actual y completo, estableciendo la posibilidad de abordajes experimentales básicos y clínicos desde un enfoque genético–molecular.

## Contribuciones de los Autores

ADMA, KPML, DBMS y AMMS: redacción del manuscrito, adquisición y análisis de información; LGV y GLT: concepto y diseño, redacción del manuscrito, adquisición y análisis de información, revisión crítica del manuscrito por contenido. Todos los autores leyeron y aprobaron el manuscrito final. Todos los autores han participado lo suficiente en el trabajo y han acordado ser responsables de todos los aspectos de este.

## Aprobación Ética y Consentimiento Informado

No aplicable.

## Agradecimientos

No aplicable.

## Financiación

Esta investigación no recibió financiación externa.
